# Programmed intermittent adductor hiatus block enhances early recovery after total knee arthroplasty: a randomized controlled trial

**DOI:** 10.1186/s42836-026-00390-x

**Published:** 2026-04-17

**Authors:** Zhanghuan Tian, Kaihua He, Jun Dong, Zhiqiao Wang, Jiaxing Chen, Wenlong Yan, Wei Ran

**Affiliations:** 1 Nursing Department, The First Affiliated Hospital of Chongqing Medical University, Chongqing, 400042 China; 2 Department of Anesthesiology, The First Affiliated Hospital of Chongqing Medical University, Chongqing, 400016 China; 3 Department of Orthopedics, The First Affiliated Hospital of Chongqing Medical University, Chongqing, 400016 China

**Keywords:** Programmed intermittent infusion, Adductor hiatus block, Total knee arthroplasty, Knee range of motion, Muscle strength, Post-operative recovery

## Abstract

**Background:**

Multimodal analgesia based on ultrasound-guided regional block is widely used after total knee arthroplasty (TKA). The goal of this study was to investigate the analgesic efficiency and knee motor function of programmed intermittent infusion combined with adductor hiatus block in total knee arthroplasty.

**Methods:**

This prospective randomized controlled trial was approved by the Medical Ethics Committee of the First Affiliated Hospital of Chongqing Medical University (ethical approval number: 2024-302-01) and was registered in the Chinese Clinical Trial Registry (http://www.chictr.org.cn, ChiCTR2400090031); the study was conducted from October 2024 to March 2025. A total of 148 patients undergoing unilateral total knee arthroplasty with general anesthesia were assigned to the continuous adductor canal block (CACB) group (G1, *n* = 50), the continuous adductor hiatus block (CAHB) group (G2, *n* = 50), or the programmed intermittent adductor hiatus block (PIAHB) group (G3, *n* = 48). The main outcome was the active flexion angle of the knee joint. The secondary outcomes were performance on the timed up-and-go (TUG) test; the muscle strength of the quadriceps femoris, ankle dorsiflexors, and metatarsal flexor; and Visual Analogue Scale (VAS) scores of anterior and posterior sides of the knee at rest and during active 30-degree flexion.

**Results:**

The PIAHB group had a significantly greater active knee flexion angle than the CAHB and CACB groups on the 1st, 2nd, and 3rd post-operative days (*F* = 14.313, *p* < 0.001; *F* = 16.793, *p* < 0.001; and *F* = 18.097, *p* < 0.001, respectively); the TUG times in the PIAHB group were shorter than those in the CAHB and CACB groups on the 1st and 2nd post-operative days (*F* = 26.059, *p* < 0.001) (*F* = 18.102, *p* < 0.001), but there was no difference in TUG test results on the 3rd post-operative day. There was no significant difference in the muscle strength of lower limb; VAS scores of the posterior side of the knee at rest and during active flexion were significantly lower in the PIAHB group than in the CAHB and CACB groups (*F* = 5.860, *p* = 0.004; *F* = 80.015, *p* < 0.001), but there was no difference in the VAS scores of the anterior side of the knee. The number of patients receiving remedial analgesia within 72 h was reduced in the PIAHB group (*F* = 7.405, *p* = 0.030), and the consumption of ropivacaine was significantly reduced in that group (*F* = 24.995, *p* < 0.001), but there was no difference in the incidence of postoperative complications or in HSS (post-operativeHospital for Special Surgery) scores 6 months post-operatively.

**Conclusions:**

PIAHB increased the analgesic effect on the popliteal fossa without decreasing the strength of the quadriceps femoris, resulting in improved ROM on the 1st and 2nd post-operative days in patients who underwent TKA.

## Introduction

Total knee arthroplasty (TKA) is widely used to treat end-stage osteoarthritis in elderly patients, with a high incidence of moderate to severe post-operative pain, and is regarded as one of the most painful orthopedic procedures [1, 2].

Previously, the gold standard for post-operative analgesia after TKA was multimodal analgesia dominated by continuous femoral nerve block [3, 4]. However, a high-dose femoral nerve block or a high drug concentration can reduce quadriceps muscle strength and affect post-operative motor function recovery [5, 6]. Recently, researchers have proposed that the creation of anatomic block pathways could be a solution to this problem. Researchers have made significant advances in distal adductor canal block, infiltration between the popliteal artery and capsule of the knee (IPACK) block, obturator nerve branch block, and other methods in recent years. Continuous peripheral nerve blocks (PNBs) were slightly more effective in the first 24 h after surgery but were more frequently associated with motor blockade, which should be avoided [4, 7–10]. Currently, however, analgesic effectiveness and preservation of muscle strength in the anterior and posterior regions of the knee joint cannot be achieved simultaneously. As a result, it is still worthwhile to search for a better analgesic program capable of balancing muscle strength preservation and pain control.

To this end, we relocated the blockage to the level of the adductor hiatus, where the femoral artery and vein cross backward into the popliteal fossa. Drug injection in this traffic area may keep the adductor canal blocked and produce an analgesic effect on the popliteal fossa area.

Continuous administration is another important tool for controlling post-operative analgesia. Previous research has shown that programmed intermittent bolus (PIB) infusion is beneficial for labor analgesia owing to its ability to deliver a higher drug volume in a shorter period of time [11, 12]. Furthermore, some studies have shown that PIB can reduce burst pain and motor nerve block, as well as produce a good analgesic effect with little effect on muscle strength [13, 14]. It has also been reported to occur in sciatic nerve block, erector spinae muscle-level block, and thoracic paravertebral block [15–17]. Many factors influence drug distribution and diffusion, including local anesthetic volume and concentration, puncture space, infusion speed, and tissue compliance [18]. In vivo and in vitro studies, however, show that faster dosing and higher dosing pressure can result in more extensive diffusion [19, 20]. As a result, we used a program-controlled intermittent administration technique to increase the administration pressure and volume per unit of time, potentially increasing drug penetration and diffusion behind the popliteal fossa.

In summary, the goal of this study was to investigate the analgesic efficiency and knee motor function of programmed intermittent infusion combined with adductor hiatus block in total knee arthroplasty.

## Methods

### Study design and patient characteristics

This single-center prospective randomized controlled study was approved by the Medical Ethics Committee of the First Affiliated Hospital of Chongqing Medical University (ethical approval number: 2024-302-01) and was registered in the Chinese Clinical Trial Registry (http://www.chictr.org.cn, ChiCTR2400090031). The research was conducted from October 2024 to March 2025. All the subjects signed informed consent before surgery.

#### Inclusion criteria

The study evaluated patients undergoing unilateral total knee arthroplasty under general anesthesia. Participants were included based on the following criteria: aged 65 to 85 years, body mass index (BMI) ranging from 18 to 30 kg/m^2^, American Society of Anesthesiologists (ASA) physical status classified as I to IV, and normal pre-operative cognitive function.

#### Exclusion criteria

Patients who had a history of knee surgery within 3 months on the other side, those who underwent spinal surgery within 6 months, those who were allergic to local anesthetics, those who had chronic pain requiring long-term opioid therapy, those who were unable to understand the visual analog scale (VAS), those who had an American Society of Anesthesiologists (ASA) physical status over IV, and those who had contraindications for peripheral nerve block.

### Sample size calculation

Preliminary experimental data from 10 patients in each group revealed that the average knee flexion angle was 45 degrees in the PIAHB group and 36 degrees in the CACB group, with a standard deviation of 10 degrees. At a 5% significance level and 90% power, the minimum sample size per group was 27; given an estimated dropout rate of 20%, we included a total of 148 patients. The necessary sample size was calculated by using PASS 15.0 (Stata Corp. LP, College Station, Texas, USA).

### Grouping, randomization, and blinding

Randomization was achieved using computer-generated numbers ranging from 1 to 148 (148 in total). Eligible patients meeting the inclusion criteria (assigned numbers 1 to 50) were allocated to the CACB group, those with numbers 51 to 100 to the CAHB group, and those with numbers 101 to 148 to the PIAHB group. Correspondingly, 148 numbered cards were prepared, each marked on the back with the group assignment (CACB, CAHB, or PIAHB). These cards were placed in an opaque bag. During each nerve block procedure, a senior anesthesiologist drew one card from the bag and performed the block according to the indicated group assignment. Post-operative analgesic parameters (with paper concealing the pump settings) were configured on the basis of the same group assignment. Research data were collected and analyzed by specialized personnel who were not involved in the block procedures or anesthesia management. Ward physicians and nursing staff remained unaware of the patients’ group allocations throughout the study.

### Anesthesia management

One day before surgery, the pre-operative evaluation was completed, and standard monitoring was performed after the patient entered the operating room. It was decided to install invasive arterial blood pressure monitoring and intravenous infusion channels. According to the group assignment, the same qualified anesthesiologist performed an ultrasound-guided nerve block after tripartite verification. Following a successful nerve block, all patients underwent total knee arthroplasty under tracheal intubation and general anesthesia. Midazolam (0.05 mg/kg), propofol (2 mg/kg), sufentanil (0.5 µg/kg), vecuronium (0.1 mg/kg), dexamethasone (10 mg), parecoxib (40 mg), and omeprazole (40 mg) were administered intravenously. Tidal volume was set to 6–8 mL/kg, respiratory rate was 12–16 times/min, inhaled oxygen concentration was 50%, and total flow was 2 L/min. Static absorption of sevoflurane, propofol, and remifentanil was used to maintain anesthesia, and the depth of Narcotrend anesthesia was maintained at D2–E1. A dose of 0.05 mg/kg vecuronium was added every 1 h, and controlled hypotension technology was used to keep blood pressure at approximately 20% of the baseline value during surgery (note: MAP 60 mmHg); the HR was kept at 50–100 beats per minute. Sufentanil (10 μg) and tropisetron (2 mg) were administered when the skin was being sutured. After the sutures were in place, the anesthesia was turned off. Ice packs were wrapped around the knee joint before being transferred to the PACU (postanesthesia care unit). Patients with Steward scores of 4 or higher were returned to the ward.

### Post-operative analgesia

All patients were given 40 mg parecoxib sodium once daily, 200 mg gabapentin three times daily, and patient-controlled nerve block analgesia (PCNA) after surgery (500 mg of ropivacaine and 250 mL of physiological saline for a total of 300 mL); Patients in the CACB and CAHB groups had the following pump settings: background dose 5 mL/h, lockout time 60 min. Patients in the PIAHB group had the following pump settings: program-controlled dose of 5 mL each time, administration speed 250 mL/h, and lockout time 60 min. If the VAS score was greater than 4, patients were given 50 mg of flurbiprofen axetil intravenously for immediate remedial analgesia.

### Surgical techniques

Both groups of patients were operated on by the same group of surgeons using a non-tourniquet technique. Three orthopedic surgeons used a minimally invasive mid-femoral approach and manually applied mixed bone cement implant technology to perform TKA (Posterior Stabilized; Zimmer Biomet, Warsaw, Indiana, USA). Both groups received a cocktail of local analgesia (formulation: 100 mg of ropivacaine, 40 mg of methylprednisolone, 1 g of tranexamic acid, and 8 drops of epinephrine) at the anterior knee capsule, suprapatellar pocket, meniscus scar, and fat pad, excluding the posterior capsule, prior to tibial and femoral implants.

### Adductor canal block and adductor hiatus block

**Fig. 1:**
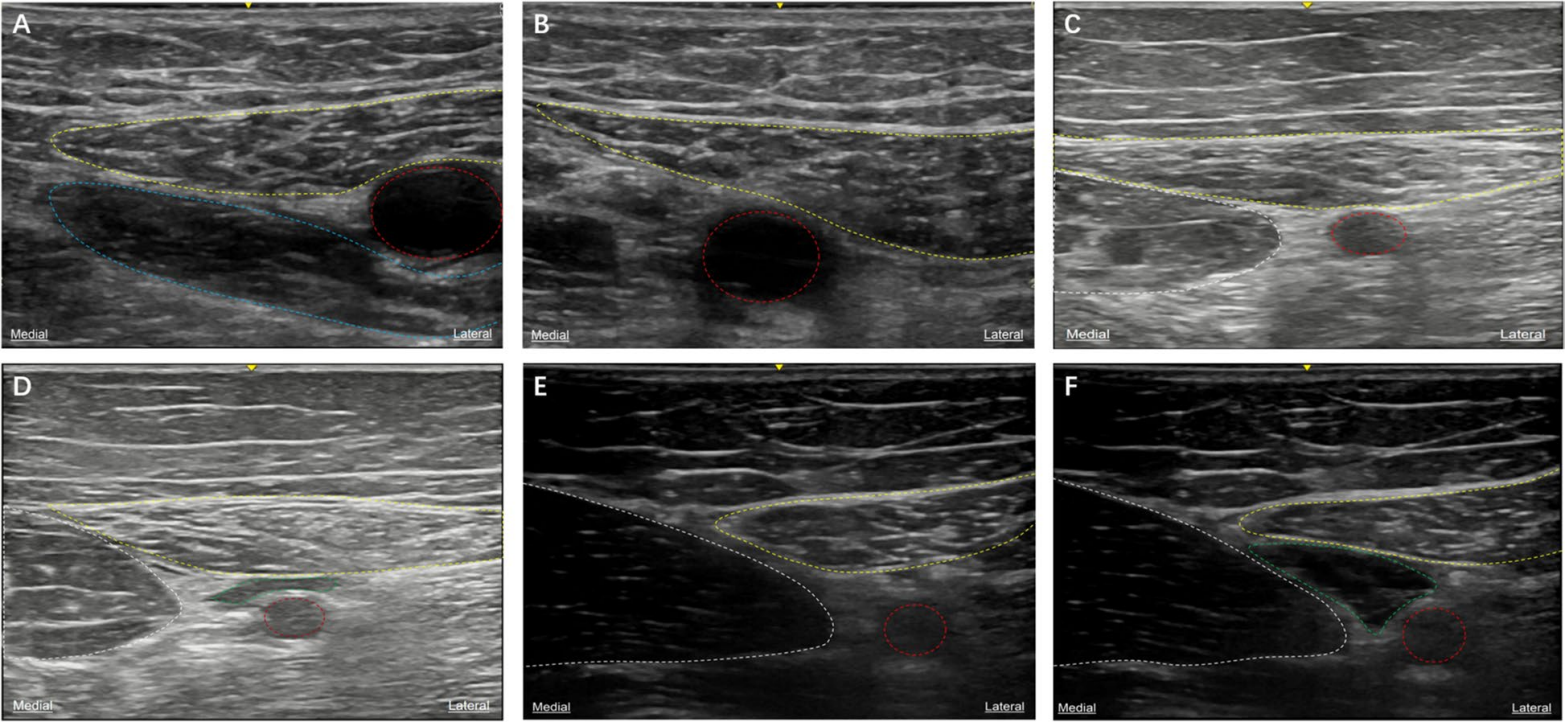
**A**: Opening of the adductor canal. **B**: Outlet of the adductor canal. **C**: Middle of the adductor canal. **D**: Middle of the adductor canal after local anesthesia. **E**: Hiatus of the adductor canal. **F**: Hiatus of the adductor canal after administration of local anesthetic. Red = femoral artery; blue = adductor longus; white = vastus medialis muscle; yellow = sartorius; green = local anesthetics

Both blocks were performed using a high-frequency linear array probe (Sonosite M-Turbo, HFL38x, 6–13 MHz) with in-plane insertion and water separation positioning techniques. In each case, 10 mL of 0.2% ropivacaine was injected after negative aspiration (no blood, air, or fluid) at a depth of 7–8 cm. Catheter fixation and the criteria for a successful block were consistent across both procedures (Figs. 1D and 1 F).

The target level differed between the two techniques. For the adductor canal block, the opening level was identified when the medial edge of the sartorius intersected the medial edge of the adductor longus; the outlet level was marked when the adductor longus vanished (Figs. 1A and 1B). The needle tip was positioned in the middle of the adductor canal (Fig. 1C).

**Fig. 2:**
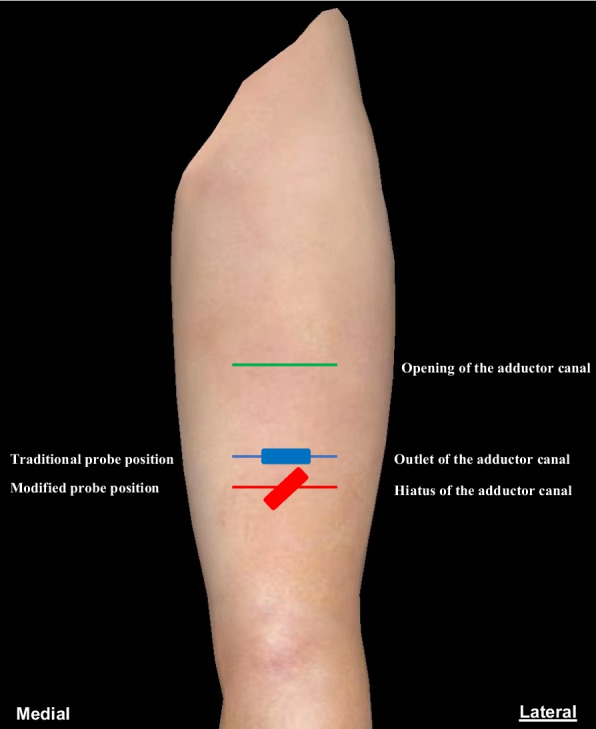
Schematic diagram of modified adductor block

For the adductor hiatus block, the insertion site was the lower level of the adductor canal hiatus, identified when the femoral artery continued distally 2–3 cm beyond the sartorius (Fig. 1E). The probe was rotated to achieve an appropriate insertion angle (Fig. 2) (This position remains anterior to the thigh, well away from the popliteal fossa, with a limited catheter insertion depth of only 2–3 cm into the hiatus; additionally, under ultrasound guidance, the catheter tip is directly visible at the adductor canal hiatus, where color Doppler demonstrates significant fluid flow during injection).

#### Main outcome measures

The main outcome was the active flexion angle of the knee joint. The angle was measured with a protractor. During the measurement, the midpoint of the protractor base was placed at the center point of the knee joint (lateral position). The bottom edge of the protractor was parallel to the middle part of the femur. When the patient actively bent the knee, the active swing arm of the protractor was positioned parallel to the middle of the tibia to measure the flexion angle of the knee joint.

#### Secondary outcome measures

(1) General information about both groups of patients was collected. (2) On the second and third post-operative days, the timed up-and-go (TUG) test time was recorded (the patient initially sat in an armchair with both hands holding a walker, with a colored tape piece stuck to the floor 3 m away from the chair. The patient stood up from the armchair, walked forward 3 m with the walker, crossed the mark, and returned to the armchair, turning around and sitting down. Patients were allowed to practice 1 to 2 times before the formal test, and the time interval from leaving the chair to sitting down again was recorded. (3) Quadriceps femoris, ankle dorsiflexor, and ankle plantar flexor muscle strength were assessed using manual muscle testing at 6, 12, 24, 48, and 72 h post-operatively. (4) VAS scores during rest and active flexion at the anterior and posterior sides of the knee joint were recorded. (5) The intra-operative opioid dosage, analgesic pump ropivacaine consumption, and the number of patients who received relief analgesia were recorded. (6) post-operative knee swelling (an increase in the mid-patella circumference by more than 3 cm compared to the pre-operative baseline was defined as knee swelling). (7) The incidence of puncture complications and adverse reactions was also recorded. (8) Knee function assessment with the Hospital for Special Surgery (HSS) scale within 6 months.

### Statistical analysis

For statistical analysis, SPSS 25.0 was used, and missing data were excluded. For intergroup comparisons of measurement data, the independent sample Student’s t test or the Wilcoxon–Mann–Whitney test was used. Mean standard deviation (Mean and SD) was used for normally distributed data, and median [interquartile range] was used for nonnormally distributed data. When variances were homogeneous, least significant difference (LSD) tests were used for subsequent pairwise comparisons; when variances were not homogeneous, Tamhane’s T2 tests were used for correction. The use rates of counting data and categorical variables, as well as the χ^2^ test or Fisher’s exact probability test, were determined using GraphPad Prism 8.3.0. (San Diego, California, USA). *p* < 0.05 was considered to indicate statistical significance.

## Results

### Experimental process

**Fig. 3:**
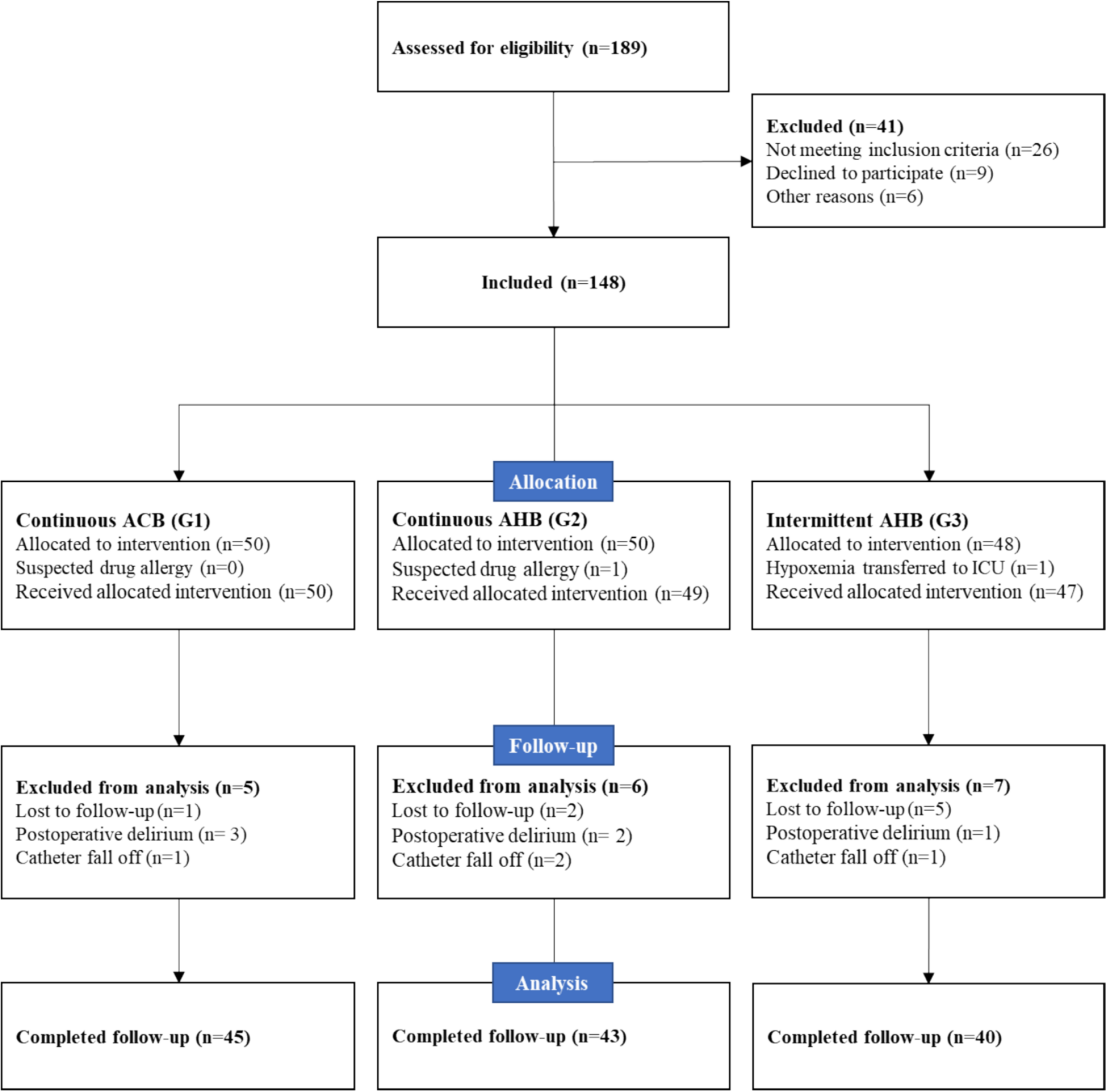
Consolidated Standards of Reporting Trials flow diagram. ACB indicates adductor canal block, and AHB indicates adductor hiatus block

In total, 148 of the 189 patients enrolled in the study between October 2024 and March 2025 were randomly assigned. Twenty patients were unable to complete the experimental procedure for a variety of reasons, including one who was suspected of being allergic to drugs prior to surgery and another who was transferred to the intensive care unit due to respiratory failure. Following surgery, eight patients were lost to follow-up at critical time points, resulting in incomplete data; six patients developed acute post-operative delirium, and four patients lost their PCNA catheter. Finally, the trial and data analysis included 128 patients (Fig. 3). We excluded missing data from the statistical analysis.

### Multiple comparison correction

For post hoc pairwise comparisons, the Bonferroni correction method was employed to strictly control the familywise error rate and reduce the risk of Type I error. Specifically, when the differences among the CACB, CAHB, and PIAHB groups were compared at all key time points, the significance level was adjusted to α = 0.05/3 ≈ 0.0167. After reanalysis, the *p*-values for all planned pairwise comparisons were found to be below this corrected threshold. Therefore, the between-group differences were considered statistically significant.

There were no differences between the groups in terms of age, BMI, gender, ASA physical status score, comorbidities, or intra-operative information (Table 1).

**Table 1:** Baseline data

	**G1 (*n* = 45)**	**G2 (*n* = 43)**	**G3 (*n* = 40)**	***F* value**	***p*-value**
**Patient characteristics**					
Age (y)	71 (63 to 77)	66 (60 to 74)	70 (59 to 75)	1.683	0.190
BMI (kg/m^2^)	25.73 ± 2.85	25.28 ± 2.72	25.21 ± 3.55	0.369	0.692
Sex				χ = 1.385	0.50
Female	35 (77.8%)	33 (76.7%)	27 (67.5%)		
Male	10 (22.2%)	10 (23.3%)	13 (32.5%)		
**ASA physical status**				χ = 1.153	0.886
II (*n*, %)	28 (62.2%)	29 (67.4%)	28 (70.0%)		
III (*n*, %)	13 (28.9%)	12 (27.9%)	9 (22.5%)		
IV (*n*, %)	4 (8.9%)	2 (4.7%)	3 (7.5%)		
**Comorbidities**					
Hypertension (*n*, %)	16 (35.6%)	14 (32.6%)	15 (37.5%)	χ = 0.227	0.893
Chronic heart disease (*n*, %)	10 (22.2%)	7 (16.3%)	6 (15.0%)	χ = 0.875	0.646
Diabetes (*n*, %)	15 (33.3%)	10 (23.3%)	12 (30.0%)	χ = 1.121	0.571
**Intra-operative information**					
Duration of operation (min)	69.67 ± 26.35	66.02 ± 18.91	62.88 ± 13.32	1.170	0.314
Duration of anesthesia (min)	121.89 ± 13.45	119.77 ± 14.76	117.20 ± 15.47	1.101	0.336
Propofol dosage (mg)	423.78 ± 80.35	401.40 ± 85.57	393.25 ± 68.18	1.744	0.179
Sufentanil dosage (mg)	41.00 ± 4.84	41.51 ± 4.82	39.88 ± 6.04	1.052	0.352
Remifentanil dosage (mg)	0.869 ± 0.22	0.91 ± 0.26	0.88 ± 0.25	0.346	0.708
Vecuronium (mg)	9.42 ± 1.12	9.23 ± 1.13	9.13 ± 1.18	0.744	0.477
Infusion volume (mL)	1320 ± 231	1304 ± 226	1318 ± 242	0.057	0.944

Categorical variables are reported as frequencies (%) and were analyzed by the *χ*^*2*^ test. Normally distributed variables are reported as the mean ± standard deviation and were analyzed by ANOVA, and the SNK-Q test was used for pairwise comparisons between groups. Nonnormally distributed variables are reported as the median [interquartile range] and were analyzed by the Kruskal–Wallis test. *p < 0.05. BMI indicates body mass index. ASA means American Society of Anesthesiologists

#### Main outcome measures

**Fig. 4:**
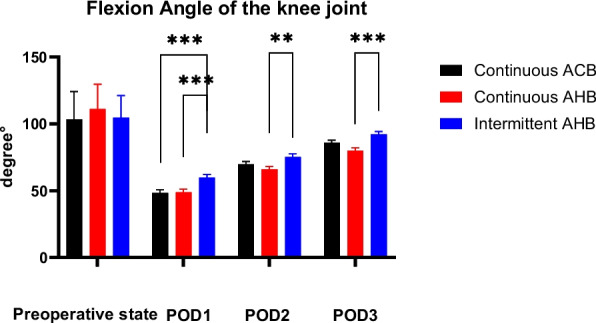
Flexion angle of the knee joint on the 1st, 2nd, and 3rd post-operative days. POD indicates post-operative day, ACB indicates adductor canal block, and AHB indicates adductor hiatus block

The active flexion angle of the knee joint significantly increased in the PIAHB group on the 1st, 2nd, and 3rd post-operative days (*F* = 14.313, *p* < 0.001; *F* = 16.793, *p* < 0.001; *F* = 18.097, *p* < 0.001, respectively). However, there was no difference pre-operatively (*F* = 2.187, *p* = 0.117) (Fig. 4).

### Secondary outcome measures

**Fig. 5:**
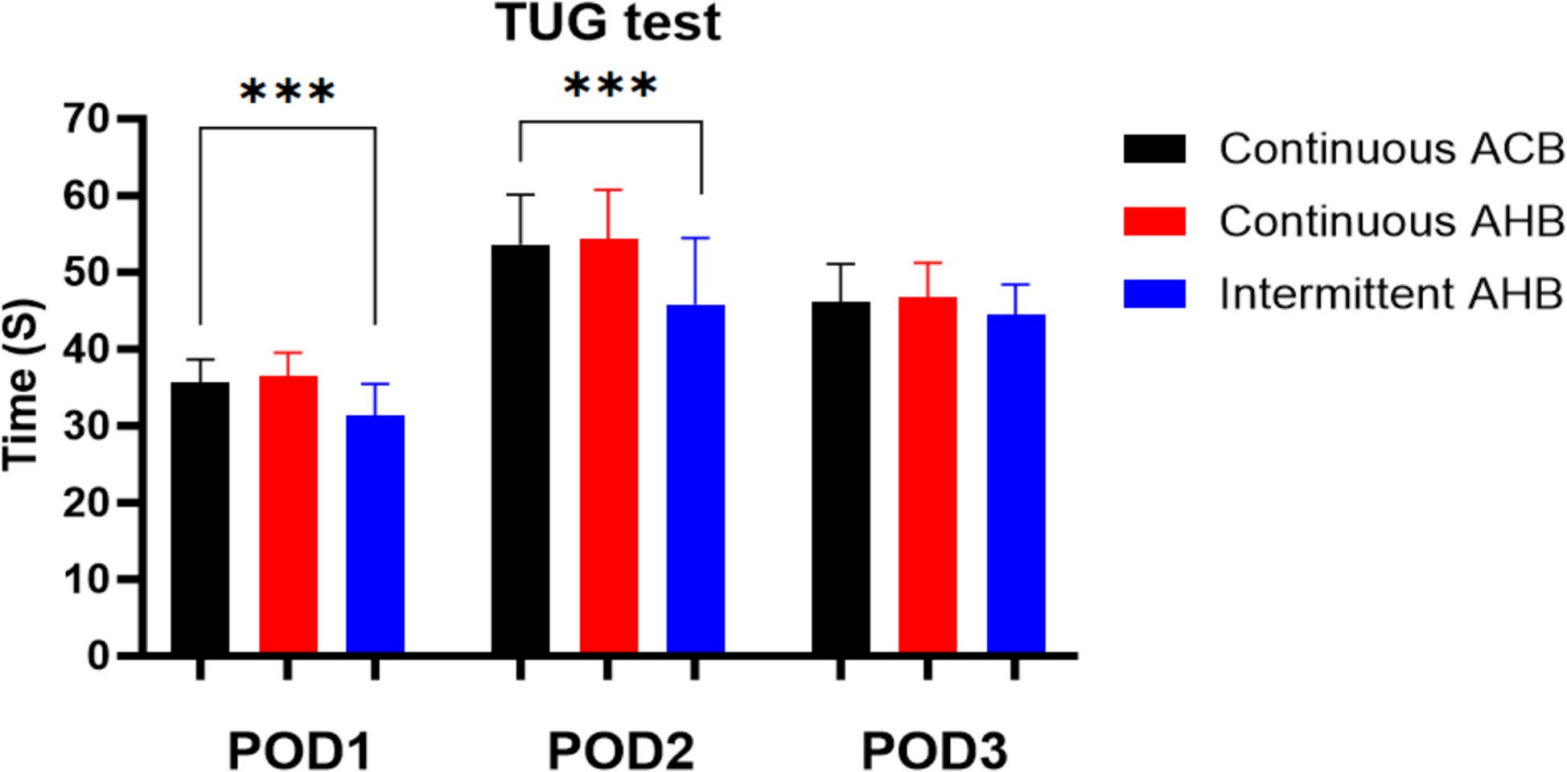
TUG tests on the 2nd and 3rd post-operative days. POD indicates post-operative day, TUG indicates the time up-and-go test, ACB indicates adductor canal block, and AHB indicates adductor hiatus block

**Fig. 6:**
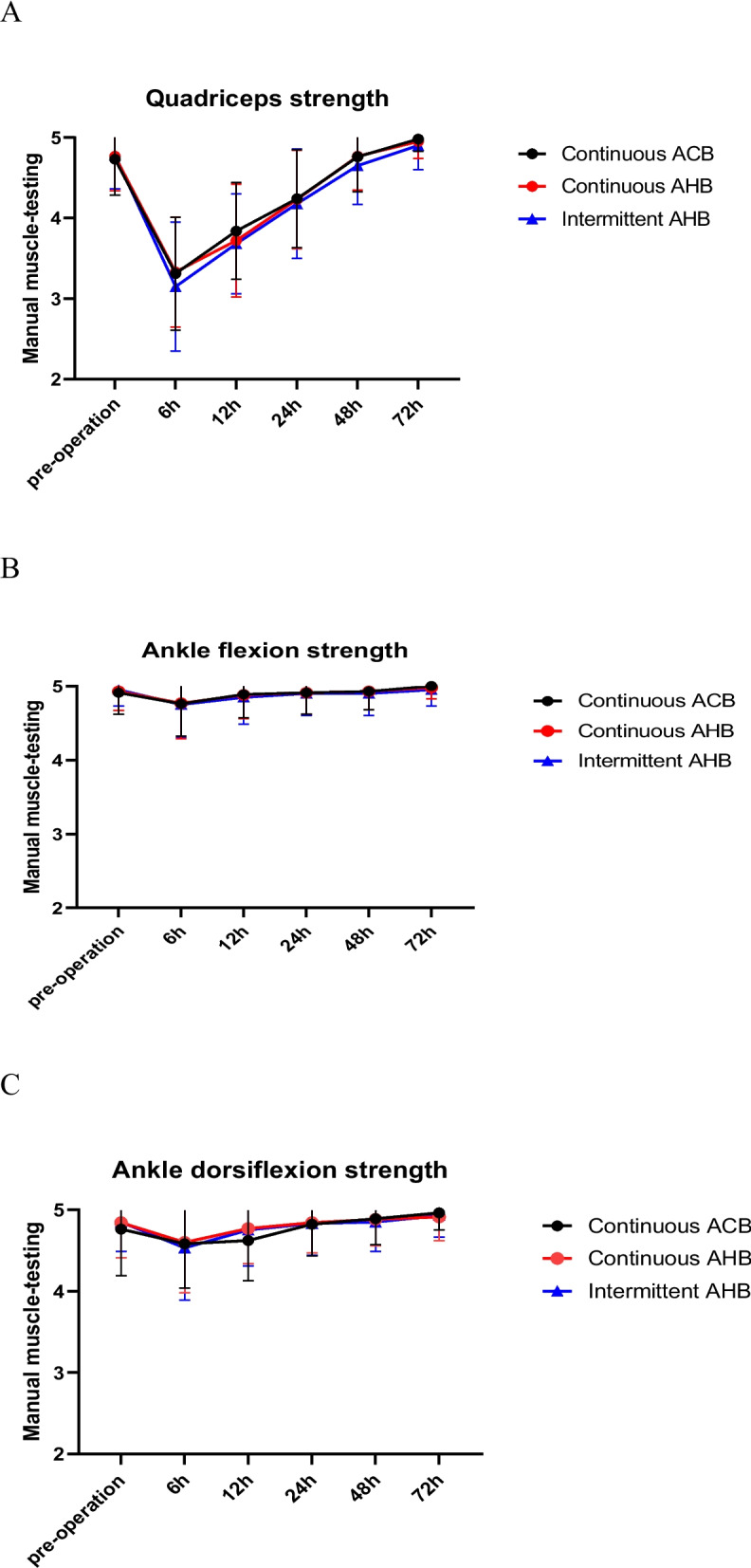
Quadriceps muscle, ankle flexion, and ankle dorsiflexion strength changes within 72 h after surgery (*F* = 1.014, *p* = 0.366; *F* = 0.061, *p* = 0.941; *F* = 0.139, *p* = 0.871). ACB indicates adductor canal block, and AHB indicates adductor hiatus block

**Fig. 7:**
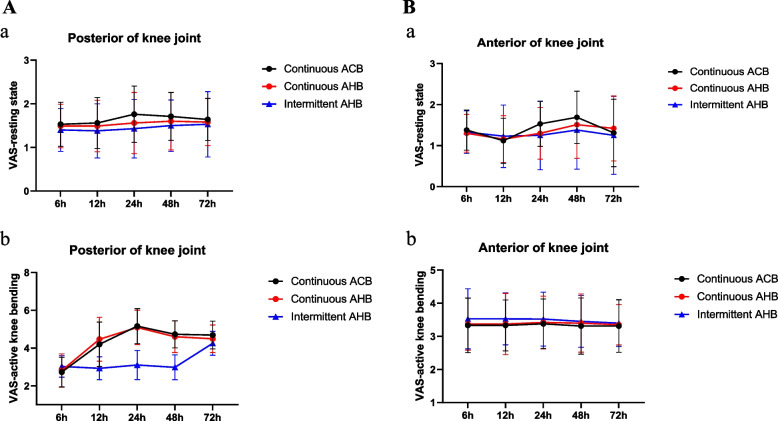
Visual analog scale scores of the posterior knee joint in resting (**a**) and active knee flexion (**b**) states within 72 h after surgery (*F* = 5.860, *p* = 0.004; *F* = 80.015, *p* < 0.001). LSD-t tests revealed that the posterior VAS score at rest was lower in the PIAHB group than in the CACB group (*p* = 0.001). LSD-t tests revealed that the posterior VAS score during active knee flexion was lower in the PIAHB group than in the CACB and CAHB groups (*p* < 0.001). ACB indicates adductor canal block, and AHB indicates adductor hiatus block

**Fig. 8:**
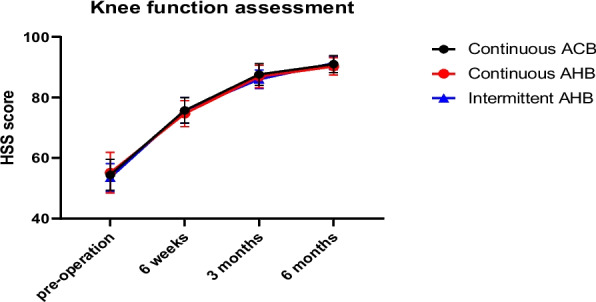
Visual analog scale scores of the anterior knee joint in resting (**a**) and active knee flexion (**b**) states within 72 h after surgery (*F* = 1.359, *p* = 0.261; *F* = 1.126, *p* = 0.328). ACB indicates adductor canal block, and AHB indicates adductor hiatus block

**Fig. 9:**
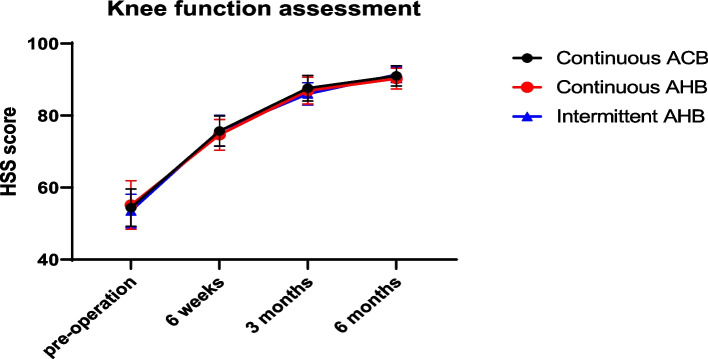
HSS score within 6 months post-operatively. HSS score indicates Hospital for Special Surgery score, ACB indicates adductor canal block, and AHB indicates adductor hiatus block

(1) TUG test results in the PIAHB group were shorter than those in the CAHB and CACB groups on the 1st and 2nd days after surgery (35.60 ± 3.07S vs. 36.40 ± 3.09S vs. 31.33 ± 4.08S, *F* = 26.059, *p* < 0.001) (53.64 ± 6.46S vs. 54.40 ± 6.36S vs. 45.80 ± 8.63S, *F* = 18.102, *p* < 0.001), but there was no difference on the 3rd day after surgery (46.11 ± 4.95S vs. 46.74 ± 4.49S vs. 44.55 ± 3.83S, *F* = 2.623, *p* = 0.077) (Table 2) (Fig. 5). (2) There were no significant differences in quadriceps strength, ankle flexion strength, or ankle dorsiflexion strength between the two groups (*F* = 1.014, *p* = 0.366; *F* = 0.061, *p* = 0.941; *F* = 0.139, *p* = 0.871) (Fig. 6). (3) The VAS score for the posterior position in the resting state and passive bending of the knee was significantly lower in the PIAHB group than in the other groups (*F* = 5.860, *p* = 0.004; *F* = 80.015, *p* < 0.001) (Fig. 7), but there were no differences in anterior VAS score between the resting state and passive bending state (*F* = 1.359; *p* = 0.261; *F* = 1.126; *p* = 0.328) (Fig. 8). (4) The number of patients who received remedial analgesia within 72 h in the PIAHB group was reduced to 10 (22.2%) vs. 16 (37.2%) vs. 5 (12.5%) (*F* = 7.045, *p* = 0.030), and the consumption of ropivacaine in the PIAHB group was significantly reduced (450.70 ± 18.4 mg vs. 447.8 ± 19.8 mg vs. 422.4 ± 21.9 mg, *F* = 24.995, *p* < 0.001) (Table 3). (5) There were no significant differences in patients with post-operative knee swelling and other block-related data and incidence of adverse effects between groups (Table 3). (6) There was no difference in HSS assessment within 6 months after surgery (*F* = 0.633, *p* = 0.533) (Fig. 9).

**Table 2:** Motor function assessment

	**G1 (*n* = 45)**	**G2 (*n* = 43)**	**G3 (*n* = 40)**	***F* value**	***p*-value**
Range of motion (degrees)				7.321	0.001*
POD1	50 (40 to 60)	50 (30 to 60)	60 (50 to 68)	6.778	0.002*
POD2	70 (60 to 76)	70 (45 to 80)	78 (70 to 80)	4.449	0.014*
POD3	90 (80 to 95)	90 (60 to 95)	90 (90 to 95)	8.205	< 0.001*
TUG test (POD1, s)	35.60 ± 3.07	36.40 ± 3.09	31.33 ± 4.08	26.059	< 0.001*
TUG test (POD2, s)	53.64 ± 6.46	54.40 ± 6.36	45.80 ± 8.63	18.102	< 0.001*
TUG test (POD3, s)	46.11 ± 4.95	46.74 ± 4.49	44.55 ± 3.83	2.623	0.077

Categorical variables are reported as frequencies (%) and were analyzed by the *χ*^*2*^ test. Normally distributed variables are reported as the mean ± standard deviation and were analyzed by ANOVA, and the SNK-Q test was used for pairwise comparisons between groups. Nonnormally distributed variables are reported as the median [interquartile range] and were analyzed by the Kruskal–Wallis test. *p < 0.05. POD indicates post-operative day, and TUG indicates the time up-and-go test

**Table 3:** Block-related data and adverse effects

	**G1 (*n* = 45)**	**G2 (*n* = 43)**	**G3 (*n* = 40)**	***F* value**	***p*-value**
Remedial analgesia within 72 h (*n*, %)	10 (22.2%)	16 (37.2%)	5 (12.5%)	7.045	0.030*
Ropivacaine consumption (mg)	450.70 ± 18.4	447.8 ± 19.8	422.4 ± 21.9	24.995	< 0.001*
Fall down (*n*, %)	0 (0%)	0 (0%)	0 (0%)		> 0.999
Knee swelling (*n*, %)	17 (37.8)	14 (32.6%)	12 (30.0%)	0.605	0.739
Local anesthetic leakage (*n*, %)	2 (4.4%)	2 (4.6%)	1 (2.5%)	0.309	0.857
Catheter detachment (*n*, %)	1 (2.2%)	1 (2.3%)	1 (2.5%)	0.007	0.996
Articular cavity infection (*n*, %)	0 (0%)	0 (0%)	0 (0%)		> 0.999
PONV within 72 h (*n*, %)	2 (4.4%)	5 (11.6%)	3 (7.5%)	1.583	0.453
Emergence delirium (*n*, %)	3 (6.7%)	2 (4.6%)	1 (2.5%)	0.823	0.663

Categorical variables are reported as frequencies (%) and were analyzed by the *χ*^*2*^ test. Normally distributed variables are reported as the mean ± standard deviation and were analyzed by ANOVA, and the SNK-Q test was used to compare between two groups. *p < 0.05. PONV means post-operative nausea and vomiting

## Discussion

Our study presents a novel nerve block analgesic protocol that may enhance both knee joint function and pain control immediately after total knee arthroplasty. Although muscle strength was comparable across all groups, the PIAHB group showed improved joint range of motion, suggesting that the observed motor recovery was primarily driven by pain relief rather than differences in muscle strength. Overall, this protocol offers a promising approach to achieving a better balance between analgesia and preservation of muscle strength following total knee arthroplasty.

Within-depth anatomical study and ultrasound imaging of the adductor canal, an increasing number of scholars recommend accurate location of the upper and lower orifices of the adductor canal [21] (opening: the overlap of the medial margin of the sartorius and adductor longus; outlet: the femoral artery passes through the adductor duct hiatus). Previous research revealed no statistically significant differences in total opioid consumption, visual analog scale (VAS) score, catheter insertion, or block success rate between proximal adductor duct block and midcourse block [22–24]. Blocking the middle and distal adductor canals has a weaker effect on quadriceps muscle strength [25]. Although opinions differ on the best site for ACB, the further the drug administration site is from the femoral triangle, the less influence it has on muscle strength.

Khaireddine Raddaoui et al. [26] reported that after injecting 20 mL and 40 mL of 0.375% ropivacaine into the adductor duct, local anesthesia spreading into the popliteal fossa resulted in a high rate of sensory block of the peroneal and tibial nerves, but motor block was rare, and Kohei Morozumi et al. [27] reported similar results. These findings support the findings of previous cadaver injection studies by demonstrating posterior popliteal diffusion [28, 29]. We modified the traditional adductor canal block injection site by moving it down to the level of the adductor canal hiatus to reduce the likelihood of drug diffusion to the femoral triangle after high-dose administration while retaining the possibility of drug diffusion posteriorly. These modifications may have the following consequences: The drug first diffuses in the adductor canal, causing a similar blocking effect in the distal adductor canal. Second, blocking the posterior branch of the obturator nerve through the adductor duct hiatus and blocking its impulse conduction can provide analgesia to the posterior medial side of the knee joint [30, 31]. Furthermore, by rotating the probe 45°, we improved the puncture angle and decreased the angle between the needle and the adductor canal, making catheter placement easier. Unfortunately, compared with a continuous adductor canal block, a continuous adductor hiatus block did not significantly improve the active knee flexion angle on the first day after surgery.

Another modification was to change the infusion model from continuous infusion to program-controlled intermittent infusion. Thomas T. Klumpner et al. [19] tested the pressure generated when the delivery speed of normal saline was 100, 175, 300, and 400 mL/h with 4 epidural catheters, and the results revealed that the peak pressure of the catheter increased with increasing delivery speed and that the peak pressure of the porous catheter was higher than that of the single-hole catheter. Yusuke Mazda et al. [20] reported that programmed intermittent epidural bolus (PIEB) delivery analgesia at 125 mL/h was associated with a lower incidence of hypotension in a slower dose regimen than 250 mL/h. According to an in vitro study, the infusion rate is directly correlated with the peak pressure when using a programmable intermittent bolus pump [19]. Because the injection at the adductor duct hiatus had little effect on the incidence of hypotension, we increased the programmed intermittent bolus (PIB) speed to 250 mL/h to achieve greater local pressure. The median capacity of the adductor tube in previous studies was 10.79 mL (95% CI, 10.10 to 11.52 mL) [32]. The recommended rate of continuous PCNA administration is 4–8 mL/h, while ropivacaine concentrations less than 0.2% can cause lower muscle strength inhibition [33, 34]. In summary, in this study, the initial loading dose of ropivacaine for nerve block was set at 10 mL with a concentration of 0.2%. After connecting the analgesic pump, the infusion rate was set at 5 mL per hour for both the CACB and CAHB groups, whereas the PIAHB group received 5 mL at a rate of 250 mL per hour during the first 2 min of each hour with a ropivacaine concentration of 0.17%. Based on these adjustments, the combination of programmed intermittent infusion and the adductor canal block technique achieved better pain control with less impact on muscle strength.

There was no significant anterior pain difference between the groups of patients in the resting state or the passive bending 72 h after surgery, according to the data. The VAS score of the patients in the PIAHB group in the posterior resting state and during passive bending of the knee was significantly lower than that in the other groups. The number of patients who received post-operative remedial analgesia in the PIAHB group was significantly lower than that in the CACB and CAHB groups, which was consistent with the findings of previous studies [11, 35]. The data presented above suggest that using program-controlled intermittent injection after TKA can increase the ability of drug diffusion posteriorly and produce a certain post-operative popliteal analgesic effect, providing new support for the improvement of patients’ motor function in the early post-operative period. Perhaps better analgesic effects will be observed in patients undergoing surgery with a thigh tourniquet, as the applied pressure may promote more widespread diffusion of the medication.

Intra-operative tourniquets can reduce quadriceps femoris muscle strength, which can negatively affect post-operative functional recovery [36, 37]. To avoid the effect of tourniquets on lower limb muscle strength, all patients in this study were treated with a tourniquet-free technique combined with controlled hypotension during surgery. Our findings revealed an interaction effect between the blocking mode and time on the changes in the quadriceps femoris and ankle flexion and extension strength of patients in each group following surgery. As the blocking time increased, the muscles of patients in both groups tended to decline first but then increase, indicating that TKA had a negative effect on post-operative strength in all groups. There was no significant difference between the groups. These findings were consistent with those of previous studies [38, 39], in which the adductor hiatus block preserved lower limb muscle strength similar to that of the adductor canal block. Although there was no significant difference in muscle strength among the groups, lower extremity muscle strength exceeding grade 3 (manual muscle testing) was still important for guaranteeing post-operative movement ability.

We used the TUG test to assess patients’ overall post-operative mobility. TUG tests in the PIAHB group were shorter than those in the CAHB and CACB groups on the 1st and 2nd post-operative days, but there was no difference in TUG tests on the 3rd post-operative day. The above data indicate that the PIAHB group had the best analgesic effect. The similar results on the third day are most likely due to the removal of the analgesic pump and the disappearance of the local anesthetic effect.

The IPACK block is also regarded as a valuable technique for improving popliteal pain after total knee arthroplasty. Compared with the IPACK block, the advantage of the PIAHB technique lies in its ability to achieve a broader block with a single injection, while the injection site is relatively farther from the surgical area, which may reduce the risk of surgical site infection. Whether this technique can replace the IPACK block requires further clinical studies for confirmation.

In terms of adverse effects, catheter detachment, neuropathy, joint infection, and muscle toxicity caused by local anesthetics are common complications of peripheral nerve block [40, 41]. There was no neuropathy or knee joint infection in our study, and the number of post-operative catheter shedding patients in the CACB, CAHB, and PIAHB groups was one (2.2%), one (2.3%), and one (2.5%), respectively, which was lower than the proportion reported by Seo SS et al. [42]. This could be related to the more stable subcutaneous tunnel combined coil fixation method.

## Limitations

First, the TUG tests were performed early after surgery, when patients were still at risk of falling. Moreover, the effects of the TUG test on patients’ short- and long-term joint function are unknown. Second, we cannot obtain maximum voluntary isometric contraction (MVIC) measurement equipment for muscle strength assessment; thus, we must rely on the traditional unarmed muscle strength assessment method, which may result in some errors in the muscle strength measurement process. Third, we relocated the drug delivery site to the level of the adductor duct hiatus, bringing the puncture delivery site closer to the incision. Despite the fact that all nerve block procedures were performed aseptically, the risk of infection could not be completely avoided. Fourth, we did not perform imaging observations of local anesthetic drug diffusion, which could have provided direct evidence of popliteal fossa diffusion. Finally, because we did not assess post-operative cognitive function, we cannot infer what effect programmed intermittent administration combined with modified adductor canal block had on neurocognition after total knee arthroplasty.

## Conclusions

PIAHB can enhance analgesic efficacy in the popliteal region while preserving quadriceps strength and avoiding sciatic nerve blockade. Consequently, PIAHB helps improve knee range of motion in patients after total knee arthroplasty and promotes rapid recovery of joint function.

## Data Availability

All data generated or analyzed during this study are included in this published article.
